# Evaluation of a novel portable three-dimensional scapular kinematics assessment system with inter and intraobserver reproducibility and normative data for healthy adults

**DOI:** 10.1186/s40634-020-00238-6

**Published:** 2020-05-13

**Authors:** Miguel Angel Ruiz Ibán, Andrea Paniagua Gonzalez, Marco Muraccini, Cristina Asenjo Gismero, Alessandro Varini, Antonella Berardi, Matteo Mantovani

**Affiliations:** 1 Unidad de Hombro y Codo, Hospital Universitario Ramón y Cajal, Cta Colmenar km 9,100, 28046 Madrid, Spain; 2 Unidad de Hombro y Codo. Hospital Fraternidad -Muprespa Habana, Madrid, Spain; 3 NCS Lab Slr, Carpi (BO), Italy; 4 Department of Electrical, Electronic and Information Engineering, University of Bologna, Bologna (BO), Italy; 5 Unidad de Hombro y Codo. Hospital FREMAP Majadahonda, Madrid, Spain

**Keywords:** Shoulder, Range of motion, Scapular dyskinesis, Scapula, Reproducibility

## Abstract

**Purpose:**

To evaluate the intra and interobserver reproducibility of a new system that assesses the three-dimensional humero-scapulo-thoracic kinematics using wearable technology in an outpatient setting. To obtain normative data with the system for scapular angular motions in three planes.

**Methods:**

The SHoW Motion 3D kinematic tracking system is a motion analysis system that uses wireless wearable non-invasive inertial-magnetic sensors to assess the three-dimensional kinematics of the shoulder girdle. The sensors are placed over the skin in the sternum, scapular spine and arm to precisely define angular motions of the humerus and the scapula with three Degrees of Freedom (DOF) for each segment.

The system was used to measure the scapular angular motions in three planes (upward/downward rotation, internal/external rotation and anterior/posterior tilt) during two shoulder full-range movements (flexion/extension and abduction/abduction) in both shoulders of 25 healthy volunteers (13 males and 12 females, mean age: 37 [standard deviation 11.1] years). In a first measuring session one examiner made two evaluations alternating with another examiner that made a third evaluation. In a second session, one week apart, the first examiner made a fourth evaluation.

A mean curve was computed from the normalized data for each measurement to obtain normative data for scapular angular kinematics. Intra and inter-observer reproducibility was evaluated using Root Mean Square Error Estimation (RMSE) and Coefficients for Multiple Correlations (CMC).

**Results:**

Both shoulders of the 25 volunteers were evaluated four times. The two hundred resulting kinematic analyses were pooled to get normative values for relations between humeral elevation angles and the three angular movements of the scapula.

The system showed at least very good (CMC > 0.90) intra and inter-observer reproducibility for scapular tilt and upward-downward rotations both in flexion and abduction. For scapular internal-external rotation the results were acceptable (CMC > 0.75) but not as good, especially for the abduction movement. RMSE calculations showed consistently good reproducibility with RSME< 4° for all three angles evaluated in flexion and abduction.

**Conclusion:**

The SHoW Motion 3D kinematic tracking system is a quick, reproducible and easy to use system for the assessment of scapular angular kinematics in healthy adults. The data obtained is similar to that obtained with other validated methods.

**Level of evidence:**

Level II.

**Clinical relevance:**

The presented system is portable, easy to use and fast. It also has good intra and inter-observer reproducibility, making it a good tool to assess objectively scapular dyskinesis in the clinical setting. The normative data obtained is consistent with previous information available.

## Introduction

The role that scapular dyskinesis (defined as “*the alteration of normal scapular kinematics*” [12]) has in shoulder pathology is often overlooked. Nevertheless the scapula and its dynamic behaviour play a key role in shoulder problems [22]. In all the spectrum of shoulder pathologies, from instability [18] to cuff pathology [13] or acromioclavicular joint injuries [19], there are alterations in the humero-scapulo-thoracic rhythm that are involved in the genesis of the problem, its development or affect the outcomes of treatment.

Probably the main obstacle to correctly identify and manage scapular dyskinesis is the difficulty to assess it in a standardized, objective manner. Imaging studies are static and fail to identify the dynamic alterations. Physical exam has also limitations, and there is general consensus that clinical evaluation is limited to identifying whether there is an altered scapular movement pattern (although these patterns resist further classification) and to a couple of clinical test [13].

A more precise scapular kinematic assessment can be obtained in an experimental setting using motion tracking systems that either use cumbersome equipment [1, 2] or invasive sensors [15]. Recently, advances in miniaturization and computing power have allowed the development of electromagnetic motion tracking systems that allow for easier, non-invasive scapular kinematics assessment [4, 9, 21] but a simple, standardized, reproducible system that can be used seamlessly in clinic during the standard work-up of most patients has not been developed yet.

The objective of this study was to evaluate the intra and interobserver reproducibility of a new scapular motion tracking system, the SHoW Motion 3D kinematic tracking system®, that is specifically designed to be easy to use in clinical practice. Also, normative data for a healthy population was obtained.

## Material and methods

### Instrumentation

**Fig. 1:**
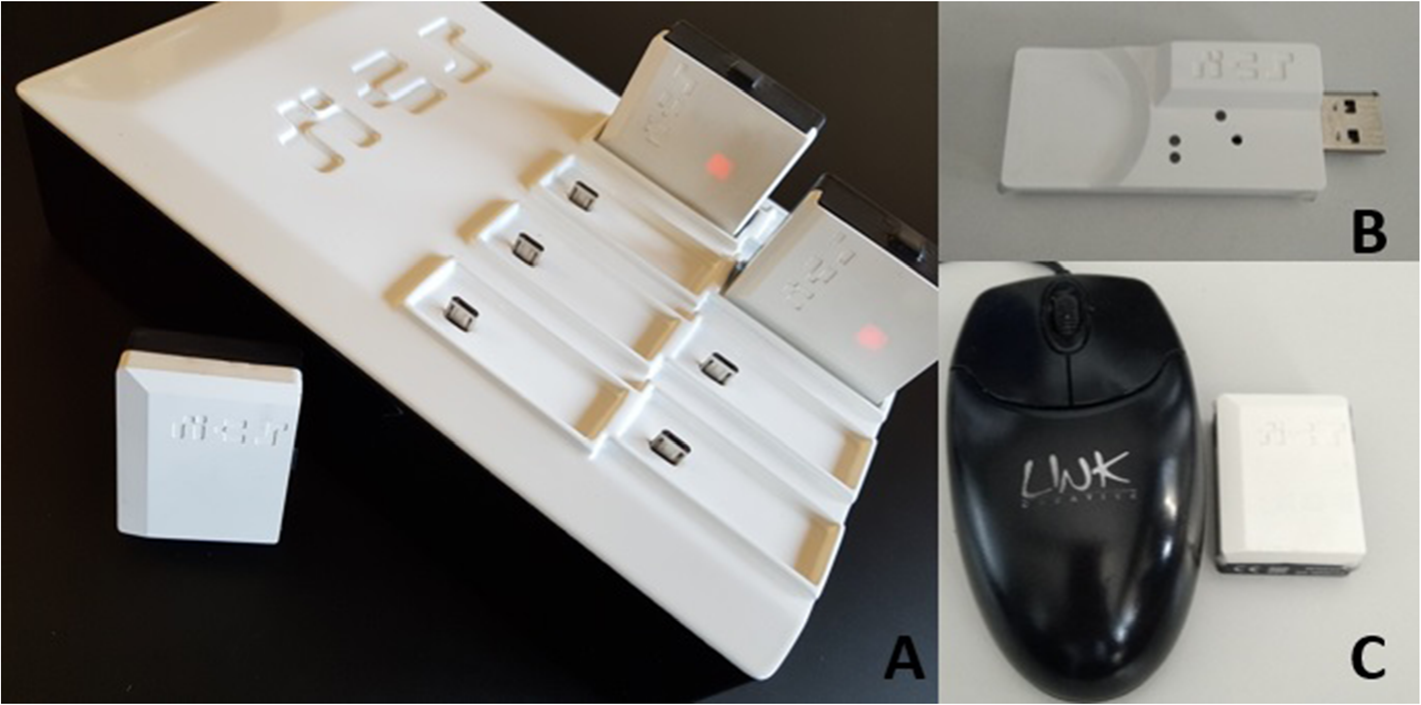
The SHoW Motion 3D kinematic tracking system is composed of a set of five sensors (here shown over a charger, **a**), a USB receiver (**b**) that plugs to any laptop and a specific software that must be installed in the laptop. The sensors have approximately the size of a matchbox (**c**). The sensors, charger straps and receiver weight less than a kilogram and fit easily in any small suitcase

**Fig. 2:**
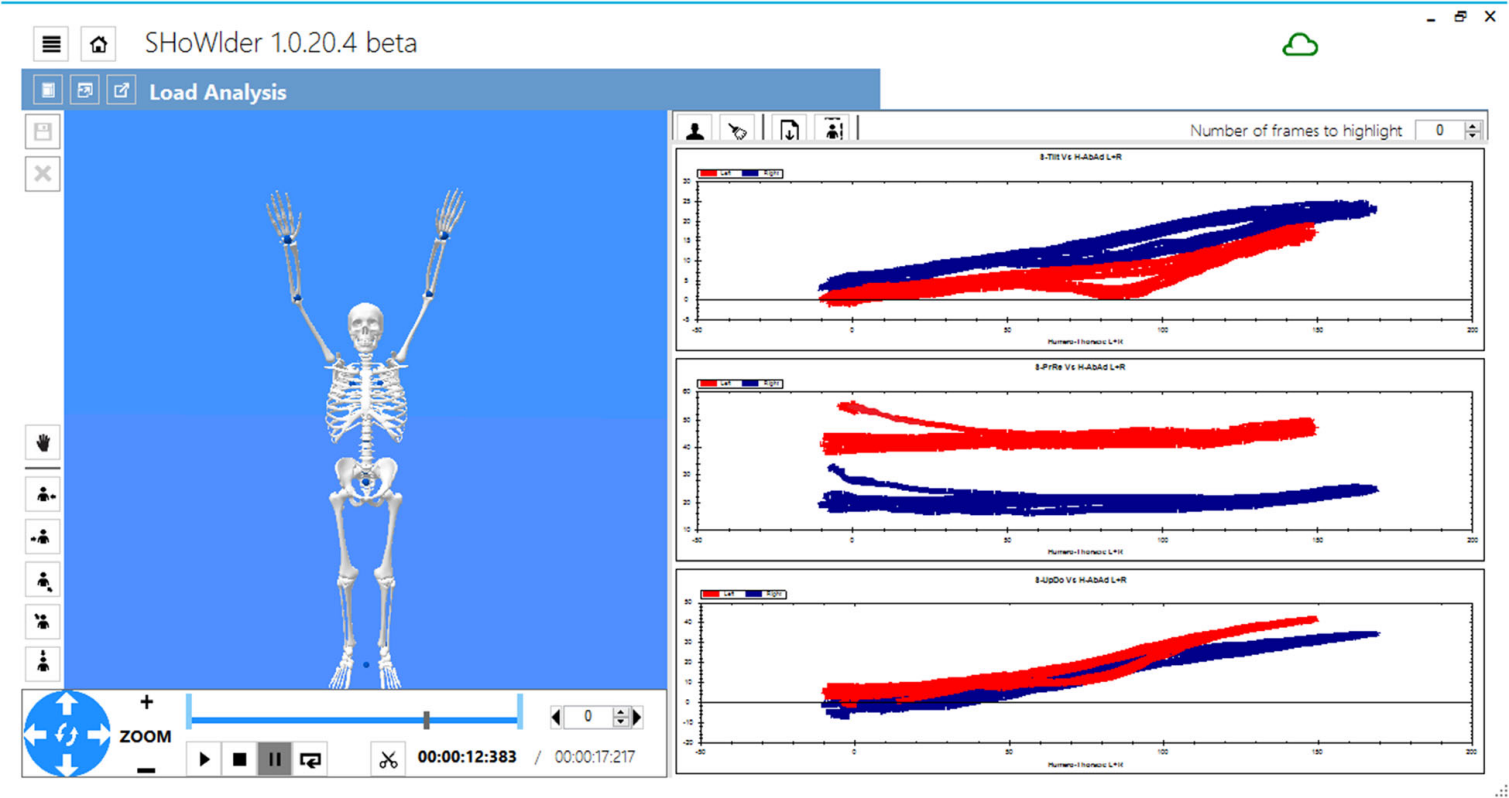
The SHoW Motion graphical interface. It shows, in real time, the angular data obtained: on the left an avatar represents the movements of the subject and can be used to confirm proper tracking. On the right, the graphs related to the scapula-humeral rhythm are displayed, for both right (blue lines) and left shoulder (red lines)

The SHoW Motion 3D kinematic tracking system (NCS Lab, Carpi, Italy) is a tool that uses motion tracking to monitor the motion pattern of the shoulder. The system includes five wireless miniature inertial measurement units (MIMU) (Fig. 1). Each MIMU provides both raw data (accelerometer, magnetometer, gyroscope) and the orientation matrix, representing the orientation of the local System of Reference (SoR) with respect to a fixed SoR. Data from each MIMU are sampled at 60 Hz and transferred wirelessly to a laptop with a proprietary software that processes the data according to a propietary biomechanical model. (Fig. 2).

First, the sensors are placed on the standing subject (one at the manubrium sterni, two on each suprascapular fossae and two over the lateral aspect of both arms) according to an standardized protocol (the INAIL Shoulder and Elbow Outpatient protocol [20]). The anatomical coordinate systems are created acquiring static reference measurements with the subject standing upright, the humerus positioned alongside the body and the elbow flexed at 90°. Then the subject, starting from a standing resting position with the arms alongside the body and the thumbs up, is asked to flex (or adduct) the shoulder until maximum elevation without pain is reached and then to return to the resting position. Each movement is repeated 7 times in a row, but only the central 5 are considered for subsequent calculations (Video 1).

**Fig. 3:**
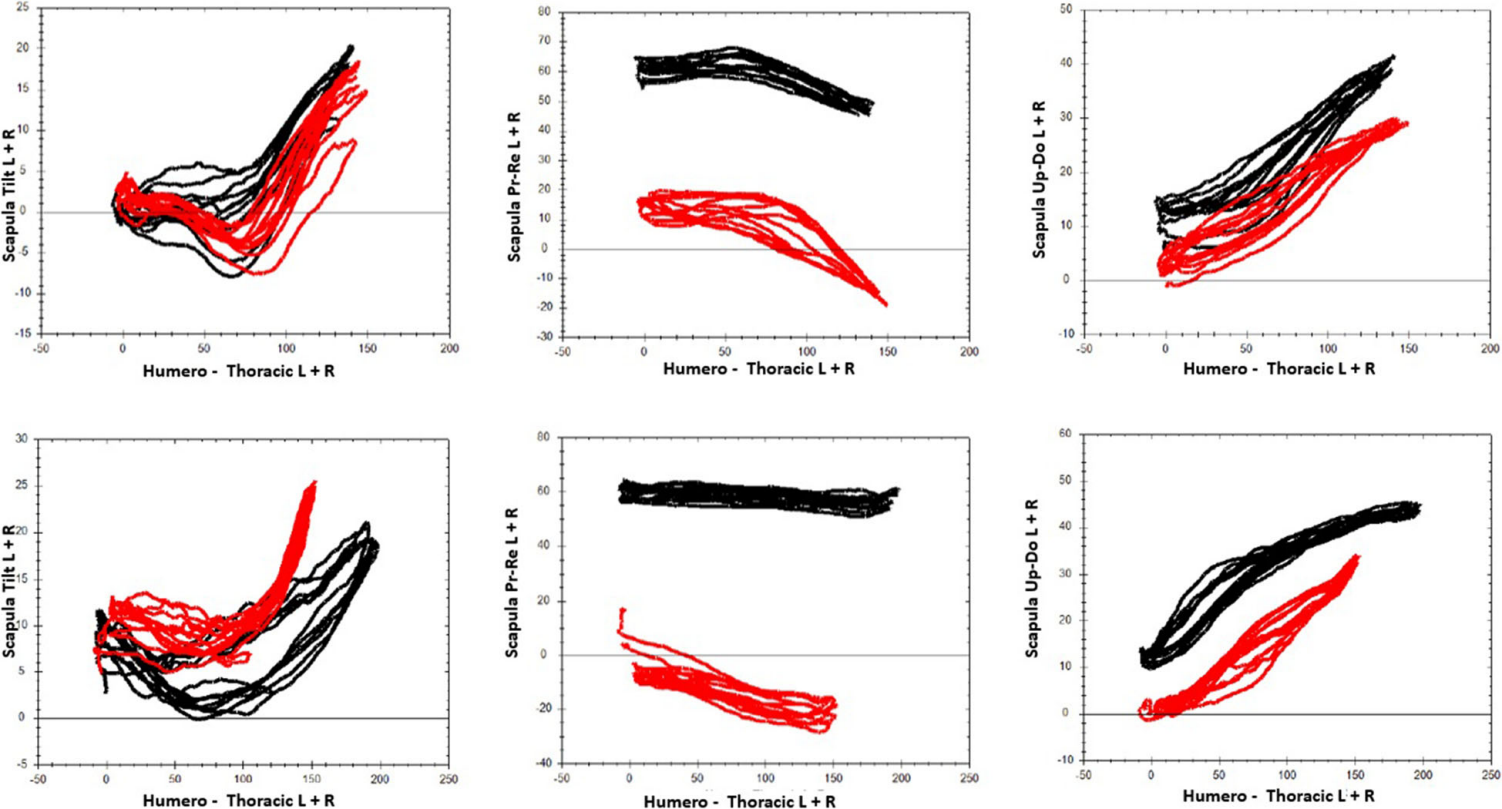
the typical output from the system. A set of six graphs that pit the variations of the three scapular angles studied (upward/downward rotation [S-UpDo], internal/external rotation [S-PrRe] and anterior/posterior tilt [S-Tilt]) against the variations in total shoulder abduction (H-AbAd) or flexion (H-FlEx). The zig-zaging lines in the graphs represent the different repetitions for each movement. There are two lines in each graph representing both arms (red lines for left side, black lines for right side). In a normal evaluation, the examiner is able to see these six graphs live as they develop

The scapular angular kinematics (SAK) are dynamically visualized in three angle–angle plots for each plane of humerus motion (flexion or abduction), in which the three scapulothoracic angular motions are plotted against humero-thoracic elevation or abduction (Figs. 2 and 3).

A standard measurement session (including time for sensor placement, calibration, measurement and sensor removal and the assessment of humero-scapulo-thoracic kinematics in the three planes during flexion/extension [FL-EX] and abduction/adduction [AB–AD] movements) can be performed by a trained operator in less than 10 min.

### Sample size calculation

Based on the recommendations of Eliasziw et al. [6] for goniometric measurements, a minimum sample size of 20 individuals (40 shoulders) was considered adequate for reproducibility analysis. A total number of 25 was estimated to control for a possible 20% loss of subjects during Coefficients of Multiple Correlation analysis [8].

### Subjects

Twenty-five healthy subjects (12 female and 13 male, mean age: 37 [standard deviation 11.1] years) agreed to participate in this research study. The inclusion criteria were: adult age (> 18 years old) and no previous history of shoulder or back pathology. The kinematic analysis of both shoulders was performed in all subjects. Since agreement between measurements does not depend on the side in which measurements are made, right and left shoulder of the subjects were considered independently for a total of 50 shoulders.

Written informed consent to participate in the study was obtained for each subject. The study was approved by the Local Institutional Review Board (IRB approval number: 207/18)

### Data collection

Two examiners took part in data collection, both had extensive experience using the system. Four measurement sessions were completed for each subject: three by examiner A (named sessions A1, A2 and A3) and one by examiner B (named session B), following the A1-B-A2-A3 sequence. The actual examiner assigned to the role of A and B was randomized for each patient. Sessions A1, B1 and A2 were planned in the same day to investigate the “same day” intra and inter-observer reproducibility. Session A3 was acquired a week later to investigate the “different day” intra-observer reproducibility. Between same-day sessions, all sensors were removed, making sure that no specific marks of the sensor placement remained, and re-applied by the new examiner, who also repeated the static calibration.

### Data processing

For the measurement of SAK, each movement (FL-EX and AB–AD) was split into an upward and a downward phase from 0° to 120°. We analysed the SAK for shoulder flexion and abduction only from 0° to 120°, to avoid data dispersion at higher humeral elevations, as previously reported [21]. Each curve was resampled to 241 equally spaced points from 0° to 120° degrees. Only the forward movement (i.e., flexion or abduction) was analysed [21].

### Prediction band for healthy subjects

A mean curve was computed from the normalized data for each subject and for each angle–angle plot. The mean curve was offset by the scapula rotation at resting position. Six angle-angle plots were obtained for each subject (three for flexion and three for abduction). The curves were averaged and merged to obtain the population prediction bands (± 2 standard deviations).

### Assessment of the protocol agreement

MATLAB software (MathWorks, Natick, MA, USA) was used to perform the statistical analysis. The intra and inter-observer agreement of the data were assessed using Coefficients of Multiple Correlation (CMC) and Root-Mean-Square Error (RMSE).

CMC are a very good alternative for reproducibility assessment of angle-angle plots such as those obtained here, as these explore the general similarities of the SAK curves, assessing the ROM, shape, offset and slope of the curves [7]. CMC analysis was conducted in two steps. First, a preliminary calculation of the CMC for intra-session consistency was performed with the data obtained in each single session, so as to exclude those subjects with high personal motion variability [10]. Second, only in subjects in which this preliminary CMC was over 0.90 (meaning they had excellent consistency in their movements), the intra- and inter-observer agreement was assessed for “same day” intra-observer agreement; “different-day” intra-observer agreement and inter-observer agreement [8]. CMC results were interpreted as follows:< 0.65, poor; 0.65–0.75 moderate; 0.75–0.85 good; 0.85–0.95, very good; 0.95–1, excellent [10, 11, 26].

RMSE is one of the most frequently used measures of the goodness of fit of generalized regression models and is a well-validated method to assess reproducibility of systems that output data in a waveform [3, 21, 25]. RMSE, calculated as defined by Parel et al. [21], were used to assess the differences between the average patterns of each side of each subject along the different sessions. RMSE were estimated for each subject, for each side and for each angle-angle plot. RMSE estimates the similarity of patterns, with low values for similar patterns and high values for different patterns. RMSE ≤4° are considered to represent good similarity between patterns [3, 21, 23].

## Results

**Fig. 4:**
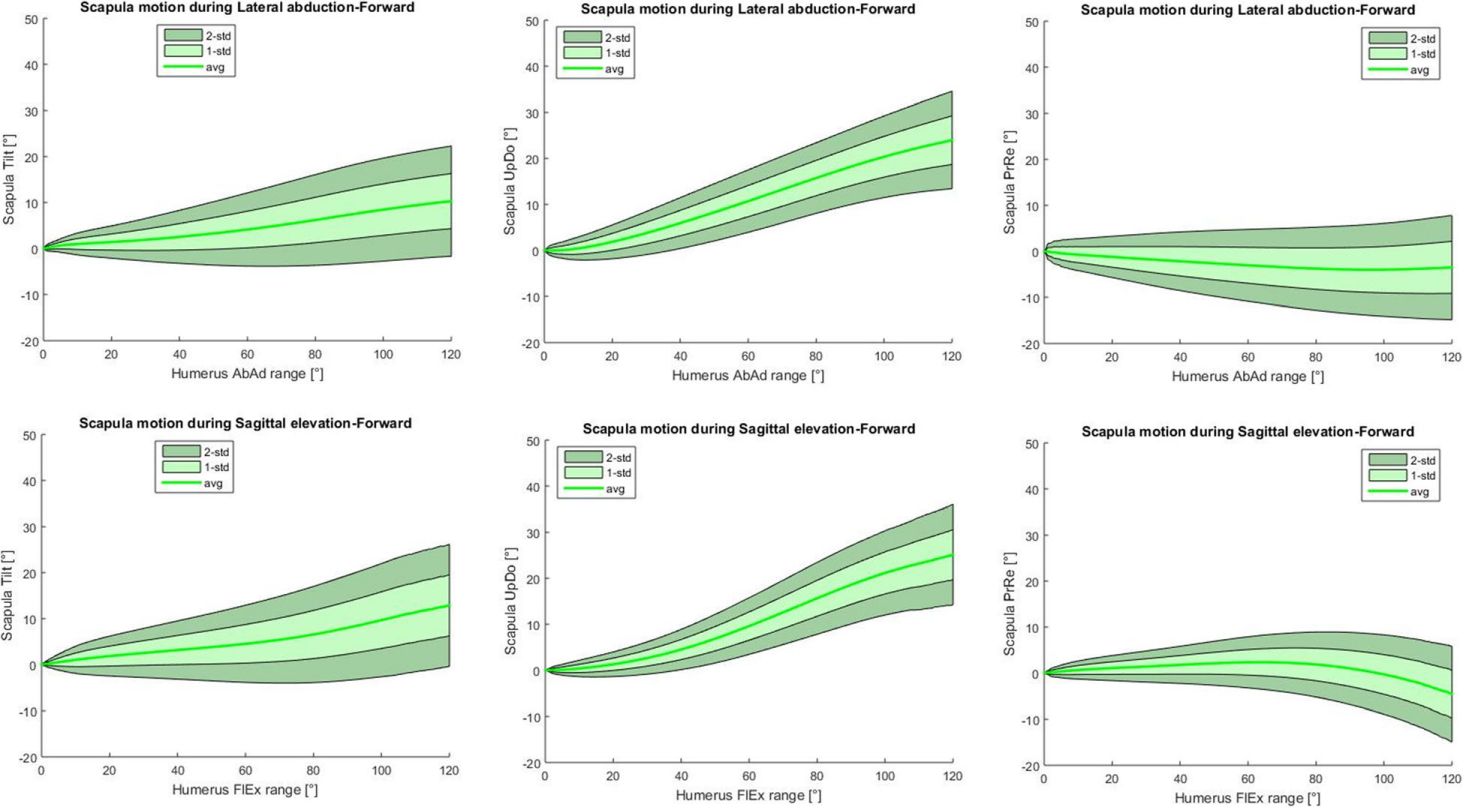
normative data for all six graphs comparing the variations of the three scapular angles studied (upward/downward rotation [Scapula UpDo], internal/external rotation [Scapula PrRe] and anterior/posterior tilt [Scapula Tilt]) against the variations in total shoulder abduction (Humerus AbAd range) or flexion (Humerus FlEx range). The green shade areas are the prediction bands for 1 SD (light green) and 2 SD (dark green)

The prediction bands representing, for each angle–angle plot, the mean curve (± 2 standard deviations) acquired over all the subjects analysed, are reported in Fig. 4. Full shoulder ROM was 170.29 ± 13.44 in abduction and 137.57 ± 9.22 in flexion. The scapular angular inclination values in the three planes for the healthy population at 30, 60, 90, 120 degrees of flexion or abduction are presented in Table 1.

**Table 1:** Representative values of the three scapular angles during flexion and abduction at 4 specific angulations (30°-60°-90° and 120°). The data is presented as mean ± standard deviation of the 200 measurements made

	Scapular internal/external rotation	Scapular upward/downward rotation	Scapular anterior/posterior tilt
30° flexion	1.41 ± 1.69	2.65 ± 1.75	2.52 ± 2.67
60° flexion	2.32 ± 2.78	9.60 ± 3.08	4.52 ± 4.20
90° flexion	0.99 ± 3.92	18.6 ± 4.26	8.00 ± 5.71
120° flexion	−4.57 ± 5.20	25.2 ± 5.44	12.9 ± 6.65
30° abduction	−1.71 ± 2.74	3.75 ± 2.35	1.89 ± 2.32
60° abduction	−3.06 ± 3.91	10.7 ± 3.40	4.12 ± 3.98
90° abduction	−3.98 ± 4.78	18.1 ± 4.10	7.35 ± 5.32
120° abduction	−3.53 ± 5.66	24.0 ± 5.29	10.3 ± 6.01

The intra-subject variability using the preliminary CMC calculation was higher than 0.90 in 81% cases for abduction and in 77% cases for flexion. Cases with lower values were excluded from the computation of the inter and intra-operator CMC. The values for all three CMC and for each scapula rotation are presented in Tables 2 and 3. The system showed excellent or very good reproducibility for most measurements, except for scapular internal/external rotation in abduction.

**Table 2:** CMC values for reproducibility of the scapular angles during shoulder flexion. The data is presented as mean ± standard deviation. Most measurement presented very good reproducibility (CMC > 0.75) with moderate reproducibility (CMC between 0.65 and 0.75) for interobserver agreement in scapular internal-external rotation

FLEXION	Scapular internal/external rotation	Scapular upward/downward rotation	Scapular anterior/posterior tilt
“Same day” intraobserver	0.74 ± 0.18	0.95 ± 0.05	0.92 ± 0.10
“Different day” intraobserver	0.74 ± 0.21	0.94 ± 0.06	0.91 ± 0.09
Interobserver	0.68 ± 0.25	0.90 ± 0.09	0.89 ± 0.14

**Table 3:** CMC values for reproducibility of the scapular angles during shoulder abduction. The data is presented as mean ± standard deviation. Most measurement presented very good reproducibility (CMC > 0.75) with acceptable results for intra-observer agreement in scapular tilt and upward/downward rotation and poor results for inter-observer agreement in scapular internal-external rotation

ABDUCTION	Scapular internal/external rotation	Scapular upward/downward rotation	Scapular anterior/posterior tilt
“Same day” intraobserver	0.75 ± 0.19	0.95 ± 0.05	0.89 ± 0.13
“Different day” intraobserver	0.73 ± 0.24	0.95 ± 0.05	0.91 ± 0.09
Interobserver	0.67 ± 0.29	0.90 ± 0.10	0.89 ± 0.15

**Fig. 5:**
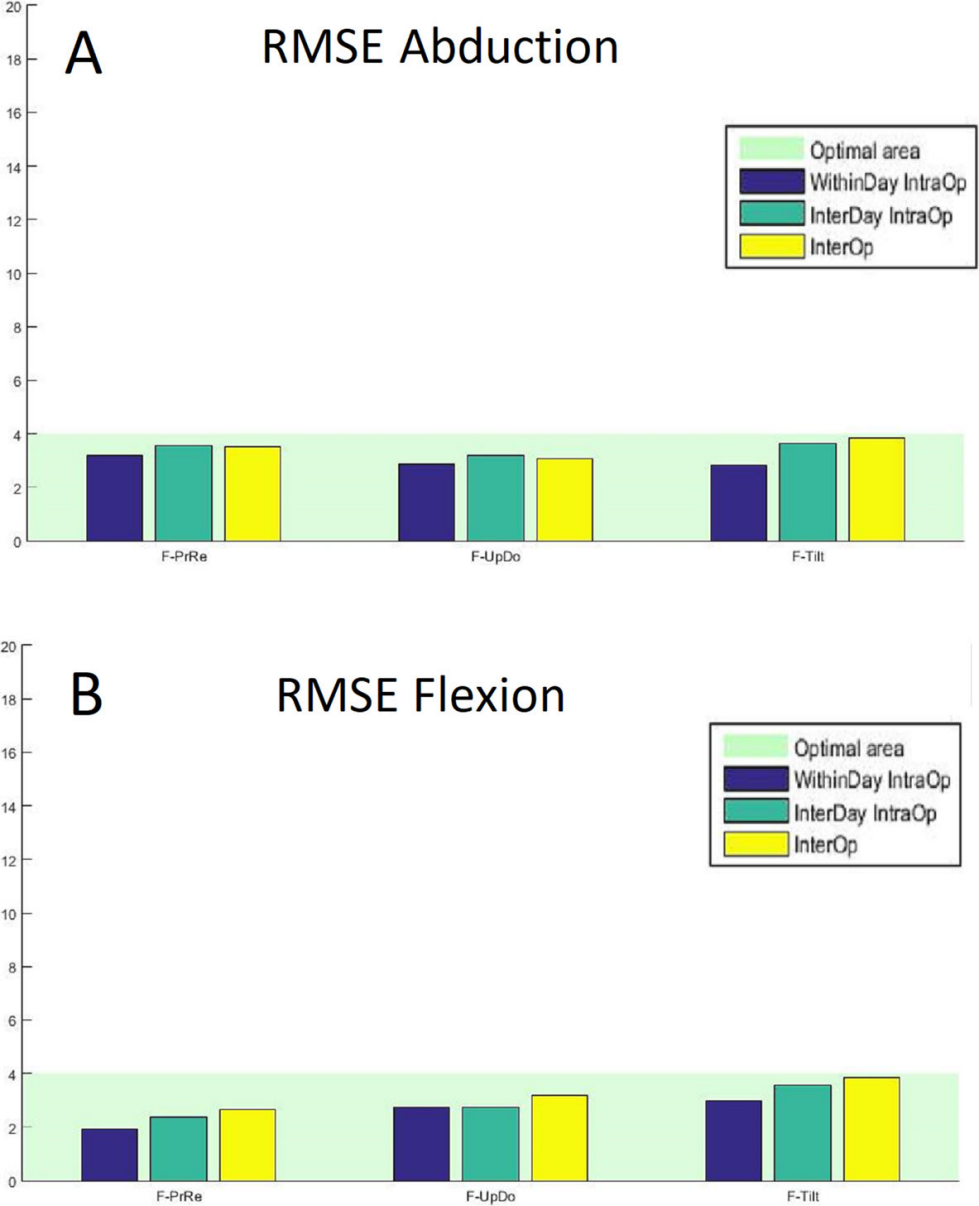
The calculated RMSE for the three reproducibility analysis (“same day” intra-observer reproducibility [WithinDay IntraOP], “different day” intra-observer reproducibility [InterDay IntraOp], and inter-observer reproducibility [InterOp]). The data is presented for all three scapular angles evaluated (upward/downward rotation [S-UpDo], internal/external rotation [S-PrRe] and anterior/posterior tilt [S-Tilt]) during flexion (**a**) and during abduction (**b**). the pale green shaded area represents RMSE values below 4°, which is considered optimal reproducibility

For both flexion and abduction tasks, the RMSE, expressed in degrees, is reported in Fig. 5 for each scapula rotation and for each comparison. The system showed good intra and inter-observer reproducibility with all measurements below 4°.

## Discussion

The most important finding of this study is that the SHoW Motion 3D kinematic tracking system is a reproducible method to assess scapular angular kinematics during flexion and adduction shoulder movements in healthy adults. Normative data from 50 shoulders regarding angular kinematics in three planes was also obtained. This system has some advantages for the clinician: it is simple to use, requires only one examiner, uses equipment that is highly portable, and it is fast enough to be used in a typical clinical setting.

Scapular dyskinesia has a significant role in many shoulder problems [13, 22], Nevertheless, there is a significant lack of proper tools to assess scapular kinematics. The only recognized mean to evaluate these in a clinical setting are visual inspection of the movement of the scapula (and comparison to the healthy contralateral side), and the scapular assistance and scapula retraction tests. Although the combination of these test has been suggested to be a good strategy to assess scapular kinematics [14], the truth is that these have a clear lack of face validity [5] and are very dependent on physician training and ability.

A lot of analysis (200 shoulder studies) were performed to present the scapular angular kinematics of a young, healthy population. The data presented in Fig. 4 is similar to the results obtained by other studies that have used optoelectronic [8, 17] or inertial-magnetic [4] systems. The variations during the flexion and abduction obtained with this novel method were consistent in range and direction with those previously reported in young and healthy population.

Two statistical procedures were used to evaluate the reproducibility of the data obtained with the new system: CMC and RMSE. CMC are often used to test the reproducibility of scapular kinematics assessment systems [2, 19, 21, 23]. RMSE is also considered an excellent tool for reproducibility assessment in shoulder biomechanics [3, 21, 25]. The reproducibility of the system presented here is similar to that of other more complex, and expensive measurement systems. Garofalo et al. [8] assessed the reproducibility of an opto-electronic system with skin markers designed to evaluate the scapular angular kinematics and found CMC values consistently around 0.95, results very similar than those obtained by our much simpler system. Parel et al. [20] evaluated the reproducibility of a cumbersome system that used non-wireless inertial-magnetic sensors to evaluate scapular kinematics and found very good intratester CMC values (over 0.90) for tilt and upward-downward rotation during abduction and flexion but the intertester reproducibility, especially for internal-external scapular rotation was unacceptable. The same group [21] also analysed the reproducibility with RMSE and obtained values consistently below 4°. Mattson et al. [17] were not able to reproducibly track scapular motion with a surface mapping system, obtaining RMSE values> 5° even when they only tested subjects with prominent scapulae.

In our study the only scapular movement that had limited interobserver reproducibility was scapular internal/external rotation during abduction. This might be because internal/external rotation of the scapula measurement is more sensible to variations in the initial positioning of the sensors on the scapular spine with respect to the other rotations. Despite of this, the results (CMC for intraobserver around 0.75 and for interobserver around 0.60) are similar to those of Parel et al. [20] using an inertial-magnetic system and compare favourably with those of Assi et al. [2] and Warner et al. [24] who, using optoelectronic systems, had difficulties in properly evaluating scapular external-internal rotations.

One limitation of this study is that there is not a direct comparison with any previously validated system. Although the system is reproducible, we cannot confirm that the measurements follow closely those found with more complicated measuring systems. Despite of this the data presented in Fig. 4 is similar to the results of others [4, 8, 17] systems, with variations during flexion and abduction consistent in range and direction with those previously reported.

Another limitation of this study is sample size, specifically regarding the obtention of normative data for healthy individuals. A sample size calculation for the reproducibility analysis was performed (something most studies in the field don’t do [16]) but the results obtained are limited in scope by the relatively young population recruited for the study.

This study has limited clinical relevance as it does not prove the system is useful in identifying and characterizing specific shoulder problems. But it provides normative data from a healthy populations and confirms that the system can be used consistently by different investigators to obtain data that is consistent in time.

## Conclusions

The SHoW Motion 3D kinematic tracking system is a quick, reproducible and easy to use system for the assessment of scapular angular kinematics in healthy adults. The data obtained is similar to that obtained with other validated methods.

## Supplementary information

**Additional file 1 Video 1**: a typical abduction and flexion measurement session using the SHoW Motion 3D kinematic tracking system.
